# The role of the EASIX score in patients with hypertension: a cross-sectional study

**DOI:** 10.1186/s43044-025-00710-7

**Published:** 2025-12-24

**Authors:** Ender Murat, Mehmet Sadık Karpat, Yusuf Öztürk, Hatice Taşkan, Ozan Köksal, Ayşe Saatcı Yaşar, Arslan Öcal, Serkan Asil, Salim Yaşar, Murat Çelik, Uygar Çağdaş Yüksel, Cem Barçın

**Affiliations:** 1 Department of Cardiology, University of Health Sciences, Gülhane Training and Research Hospital, Ankara, Turkey; 2 Department of Cardiology, University of Health Sciences, Haseki Training and Research Hospital, İstanbul, Turkey; 3 Department of Cardiology, Atatürk State Hospital, Zonguldak, Turkey

**Keywords:** Ambulatory blood pressure monitoring, Endothelial dysfunction, EASIX, Hypertension, Vascular stress

## Abstract

**Background:**

Hypertension (HTN) remains a major global health problem, and inadequate blood pressure (BP) control contributes substantially to cardiovascular risk. The endothelial activation and stress index (EASIX), derived from routine laboratory parameters, has been proposed as a marker of vascular stress. Existing studies in hypertensive populations relied on office BP measurements and included patients with multiple comorbidities. This study evaluated the association between EASIX and 24-h ambulatory BP monitoring (ABPM) in a homogeneous cohort with isolated primary HTN.

**Methods:**

This retrospective cross-sectional study included 192 adults aged 18–70 years diagnosed with HTN and undergoing 24-h ABPM. Patients were categorized into *controlled* and *uncontrolled* BP groups according to 24-h, daytime, and nighttime ABPM thresholds defined by the 2024 European Society of Cardiology guideline. The EASIX score was calculated as [lactate dehydrogenase × creatinine]/platelets and log_2_-transformed for analysis. Group comparisons, correlation analyses, multivariable logistic regression, and ROC analyses were performed.

**Results:**

The EASIX score was significantly higher in the uncontrolled BP group than in the controlled BP group. Logistic regression identified ascending aortic diameter, serum sodium, serum albumin, and log_2_ (EASIX) as independent factors associated with BP control status. EASIX demonstrated good discriminative performance in identifying hypertensive patients with inadequate BP control on 24-h ABPM.

**Conclusions:**

EASIX demonstrated good discriminative performance for identifying hypertensive patients with inadequate BP control on ABPM. These findings suggest its potential role in risk stratification, warranting validation in prospective studies.

**Graphical Abstract:**

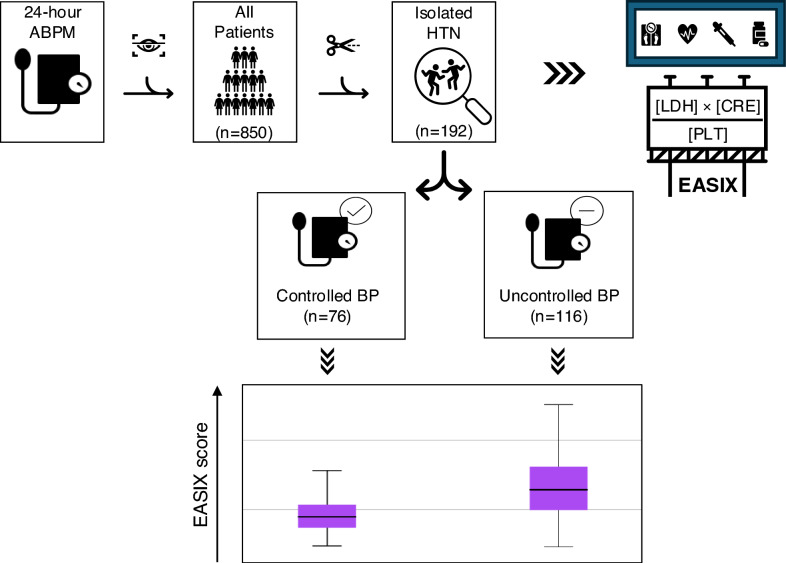

**Supplementary Information:**

The online version contains supplementary material available at 10.1186/s43044-025-00710-7.

## Background

### Hypertension (HTN)

Hypertension (HTN) is one of the most common cardiovascular conditions worldwide and remains a major driver of morbidity and mortality. Despite advances in diagnosis and therapy, a considerable proportion of individuals remain undiagnosed or achieve suboptimal blood pressure (BP) control, underscoring the need for more effective tools to support cardiovascular risk assessment [1].

HTN is diagnosed when blood pressure (BP) exceeds guideline-defined thresholds at which the expected clinical benefit of initiating therapy outweighs the potential risks [2].

Ambulatory blood pressure monitoring (ABPM) provides a more accurate assessment of BP control and overall vascular burden than office-based measurements. In addition to 24-h mean values, ABPM allows evaluation of circadian BP variation, nocturnal dipping patterns, and the detection of masked or white-coat hypertension, all of which offer important clinical insights that cannot be captured by conventional office BP readings. Multiple studies have shown that ABPM-derived averages correlate more strongly with cardiovascular and cerebrovascular outcomes and serve as more sensitive predictors of morbidity and mortality compared with clinic readings [3–9]. Given the central role of endothelial dysfunction in HTN, incorporating biochemical markers such as the endothelial activation and stress index (EASIX) may provide complementary insight into underlying vascular stress.

### Endothelial activation and stress index (EASIX)

Originally developed to quantify endothelial injury in hematologic disorders, the EASIX score has more recently been applied as an indicator of vascular damage and systemic inflammation across several cardiovascular conditions. Given that endothelial dysfunction is a core pathophysiological mechanism in HTN, we hypothesized that EASIX may similarly reflect microvascular stress associated with inadequate BP control.

Previous studies have demonstrated that vascular endothelial dysfunction contributes to the pathogenesis of acute graft-versus-host disease (aGVHD) [10, 11]. Additionally, Zeisbrich et al. reported that post-transplant thrombotic microangiopathy contributes to the recurrence of aGVHD [12]. In another study, Ruutu et al. [13] defined the diagnostic criteria for transplantation-associated thrombotic microangiopathy as elevated creatinine (CRE), increased lactate dehydrogenase (LDH), low platelet (PLT), the presence of schistocytes, and reduced haptoglobin levels [14]. Building on these findings, Luft et al. (2017) proposed the first definition of the EASIX score in a retrospective cohort of allogeneic hematopoietic stem cell transplant recipients. Because schistocyte counts and haptoglobin levels are not routinely assessed in this population, the authors refined the evaluation of endothelial injury using routinely measured parameters—CRE, LDH, and PLT count. They demonstrated an association between EASIX and aGVHD severity and defined the EASIX formula as: [LDH (U/L) × CRE (mg/dL)]/PLT (× 10^9^/L) [14].

However, existing studies primarily relied on office BP measurements and included patients with multiple comorbidities, making it difficult to isolate the relationship between EASIX and pure BP regulation. To date, no study has specifically examined EASIX in a homogeneous population with isolated primary HTN, nor has its relationship with ABPM-defined BP control been evaluated—a clinically relevant gap given ABPM’s superior prognostic value.

This study aimed to evaluate the association between EASIX and 24-h ABPM parameters in patients with isolated primary HTN. By examining a comorbidity-free cohort and applying standardized ABPM thresholds, we sought to determine whether EASIX reflects vascular stress patterns related to BP control and to provide a framework for future mechanistic and longitudinal investigations.

## Methods

### Study design and population

**Fig. 1:**
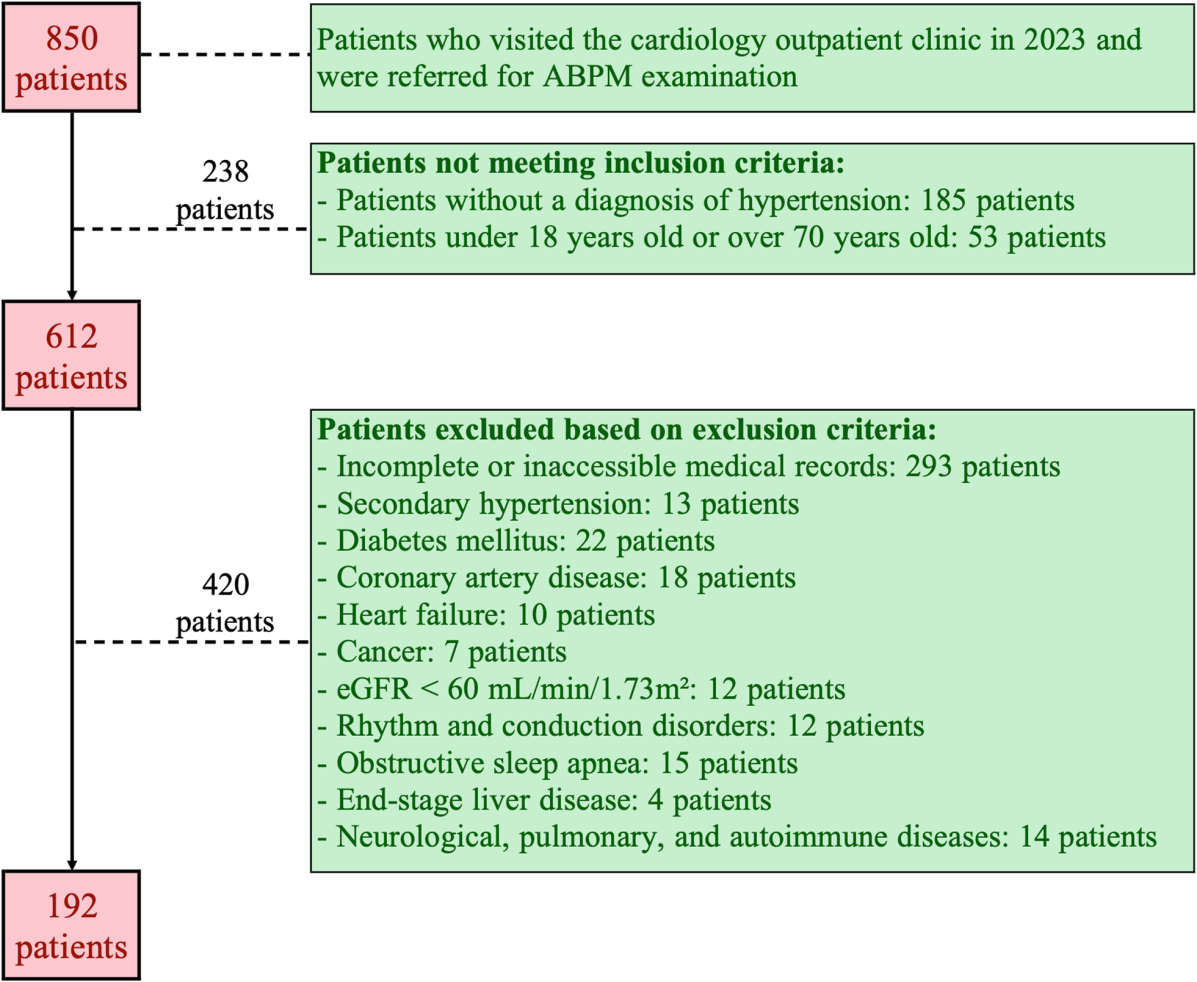
Flowchart of patient selection and study design

This investigation was designed as a retrospective cross-sectional comparative study conducted in a single tertiary cardiology center. A total of 850 patients who visited the cardiology outpatient clinic in 2023 and were referred for 24-h ABPM were initially screened. Of these, 238 individuals did not meet the inclusion criteria—185 patients had no diagnosis of HTN, and 53 were younger than 18 years or older than 70 years. The remaining 612 patients were assessed according to predefined exclusion criteria. A total of 420 patients were excluded due to incomplete or inaccessible medical records (n = 293), secondary HTN (n = 13), diabetes mellitus (n = 22), coronary artery disease (n = 18), heart failure (n = 10), cancer (n = 7), impaired renal function with eGFR < 60 mL/min/1.73 m^2^ (n = 12), rhythm or conduction disorders (n = 12), obstructive sleep apnea (n = 15), end-stage liver disease (n = 4), and neurological, pulmonary, or autoimmune diseases (n = 14). After applying all inclusion and exclusion criteria, the final study cohort consisted of 192 adult patients with isolated primary HTN who had valid ABPM recordings and complete laboratory and clinical data. Details regarding patient selection and inclusion/exclusion criteria are summarized in Fig. 1. All patients’ antihypertensive medications, statin use, and other relevant therapies were recorded.

### Ambulatory blood pressure monitoring (ABPM)

The 24-h ABPM was performed using a validated oscillometric device (Mobil-O-Graph^®^, IEM GmbH, Stolberg, Germany), programmed to obtain readings every 20 min during the daytime and every 30 min at night. Monitoring was considered valid if at least 80% of the planned measurements were successfully recorded, in accordance with the recommendations of the European Society of Cardiology (ESC) with incomplete or technically inadequate recordings were excluded from the final analysis. Daytime and nighttime intervals were defined using patient-reported sleep and wake times, ensuring individualized assessment of nocturnal BP.

Target BP thresholds (2024 ESC guideline) [2]:

- 24-h mean BP: < 130/80 mmHg
- Daytime mean BP: < 135/85 mmHg
- Nighttime mean BP: < 120/70 mmHg

Patients meeting any of the above thresholds were classified as controlled BP, while those exceeding the thresholds were classified as uncontrolled BP. Nocturnal dipping status was also recorded as contextual information (non-dipper = < 10% nighttime systolic reduction), although this was not a primary outcome.

### Echocardiographic assessment

All transthoracic echocardiographic examinations were performed using a commercially available ultrasound system (Vivid S70N, GE Healthcare, Chicago, IL, USA). Standard two-dimensional, M-mode, and Doppler measurements were acquired in accordance with current American Society of Echocardiography (ASE) guidelines [15]. Recorded parameters included left ventricular end-diastolic diameter, interventricular septal and posterior wall thicknesses, left ventricular mass index, left atrial diameter, left ventricular ejection fraction (biplane Simpson’s method), ascending aortic diameter, and systolic pulmonary artery pressure.

### Statistical analysis

Categorical variables were summarized as counts and percentages (n, %). For continuous variables, distributional characteristics were assessed, and values were reported as mean ± standard deviation for normally distributed data or median (interquartile range) for non-normally distributed data. Normality was evaluated using both the Shapiro–Wilk and Kolmogorov–Smirnov tests.

Group comparisons were performed using the independent samples t-test or one-way ANOVA for normally distributed variables, and the Mann–Whitney U test or Kruskal–Wallis H test for non-normally distributed variables. When post-hoc testing was required, Bonferroni or Dunnett’s T3 corrections were applied as appropriate. Correlation analyses were conducted using Pearson or Spearman coefficients, depending on the distribution of the data.

In accordance with prior studies utilizing the EASIX index, values were log_2_-transformed to improve distributional properties [16, 17]. Visual inspection of histograms confirmed that log_2_ transformation substantially improved the skewed distribution of raw EASIX values (Supplementary Fig S1). Subsequently, regression analyses (linear and logistic) were performed to examine associations among significant variables and to identify independent factors associated with BP control.

Patients were divided into two groups based on 24-h ABPM results, following the 2024 ESC HTN guideline thresholds [2]. Individuals whose 24-h, daytime, or nighttime systolic or diastolic BP values exceeded any of the respective guideline cut-off points were categorized as the uncontrolled BP group, whereas those who met all guideline-defined targets constituted the controlled BP group. Comparisons between these two groups were performed using appropriate parametric or nonparametric tests depending on data distribution.

An a priori power analysis was conducted using GPower version 3.1 to assess the adequacy of the sample size. The calculation targeted the detection of a difference in mean log_2_ (EASIX) values between the controlled and uncontrolled BP groups. Because no comparable ABPM-based EASIX data exist in the current literature, the effect size (Cohen’s d = 0.45) was derived from a pilot cohort of hypertensive patients evaluated at our institution. Assuming an allocation ratio of 1:1.5, a two-tailed test, and α = 0.05, the resulting statistical power was 0.92, indicating sufficient sensitivity to detect the anticipated group difference.

Candidate variables were assessed for multicollinearity using variance inflation factor (VIF) values, and those with VIF > 10 were excluded prior to model construction. A multivariable logistic regression model was then developed, incorporating age and sex as demographic covariates along with all remaining candidate variables.

The final model retained only predictors with p < 0.05. The discriminative performance of EASIX was evaluated using receiver operating characteristic (ROC) curve analysis, and optimal cut-off values were determined with the Youden index. Statistical significance was defined as p < 0.05 (two-tailed). All analyses were conducted using SPSS version 25.0 (IBM Corp., Armonk, NY, USA).

#### Hypotheses

*Null Hypothesis (H₀)*: No significant difference in EASIX scores between controlled and uncontrolled BP groups.

*Alternative Hypothesis (H₁)*: A significant difference exists in EASIX scores between controlled and uncontrolled BP groups.

## Results

A total of 192 patients with primary HTN who met the inclusion criteria were included in the analysis (Fig. 1). Baseline demographic characteristics, echocardiographic findings, and medication profiles are presented in Table 1. Patients classified as having uncontrolled BP on 24-h ABPM exhibited larger cardiac chamber dimensions and higher left ventricular mass index, consistent with structural remodeling associated with inadequate BP control. In contrast, left ventricular ejection fraction and systolic pulmonary artery pressure were similar between groups. Despite these echocardiographic differences, the use of antihypertensive medications—including β-blockers, calcium channel blockers, ACE inhibitors, and ARBs—did not differ significantly, suggesting that factors beyond pharmacologic therapy may contribute to suboptimal BP control.

**Table 1:** Descriptive characteristics, echocardiographic parameters, and medication use by BP control status

Characteristics	Group		*p*-value	
	Controlled BP (n = 76)	Uncontrolled BP (n = 116)		
Descriptive characteristics				
Age, years, Med (IQR)	45 (23)	44 (24)		0.816^a^
Sex				** < 0.001**^**c**^
Female, n (%)	62 (53.9)	53 (46.1)		
Male, n (%)	14 (18.2)	63 (81.8)		
Cigarette				0.664^c^
Smoker, n (%)	31 (37.8)	51 (62.2)		
Non-smoker, n (%)	45 (40.9)	65 (59.1)		
Height, m, Med (IQR)	1.65 (0.14)	1.70 (0.18)		** < 0.001**^**a**^
Weight, kg, mean ± SD	73.22 ± 14.54	81.47 ± 14.61		** < 0.001**^**b**^
BMI, kg/m^2^, Med (IQR)	26.6 (6.3)	27.5 (5.2)		0.143^a^
BSA, m^2^, mean ± SD	1.82 ± 0.20	1.95 ± 0.21		** < 0.001**^**b**^
Echocardiographic parameters				
Interventricular septum thickness (mm), Med (IQR)	9 (2)	10 (2)	** < 0.001**^**a**^	
Left ventricular posterior wall thickness (mm), Med (IQR)	9 (2)	9 (1)	** < 0.001**^**a**^	
Left ventricular end-diastolic diameter (mm), Med (IQR)	43 (5)	45 (4)	** < 0.001**^**a**^	
Ascending aorta diameter (mm), Med (IQR)	30 (5)	32 (5)	** < 0.001**^**a**^	
Left atrial diameter (mm), mean ± SD	32.87 ± 3.51	34.53 ± 3.25	** < 0.001**^**b**^	
Left ventricular ejection fraction (%), Med (IQR)	65 (5)	60 (5)	0.099^a^	
Systolic pulmonary artery pressure (mmHg), Med (IQR)	15 (5)	15 (10)	0.472^a^	
Left ventricular mass index (g/m^2^), mean ± SD	67.85 ± 14.46	75.21 ± 14.36	** < 0.001**^**b**^	
Relative wall thickness, mean ± SD	0.39 ± 0.05	0.41 ± 0.05	**0.037**^**b**^	
Medication use, n (%)				
Antihypertensive agents	37 (48.7)	43 (37.1)	0.110^c^	
Calcium channel blockers	20 (26.3)	25 (21.6)		0.446^c^
Beta blockers	15 (19.7)	13 (11.2)		0.101^c^
Hydrochlorothiazide	15 (19.7)	13 (11.2)		0.101^c^
ACE inhibitors	13 (17.1)	13 (11.2)		0.243^c^
ARBs	8 (10.5)	18 (15.5)		0.323^c^
Alpha blockers	3 (3.9)	3 (2.6)		0.447^d^
Loop diuretics	0 (0)	0 (0)		
Antiplatelet agents	4 (5.3)	8 (6.9)	0.447^d^	
Statins	2 (2.6)	2 (1.7)	0.518^d^	
Anticoagulants	0 (0)	0 (0)		

Data are presented as mean ± standard deviation (SD), median (interquartile range [IQR]), or number (percentage), as appropriate. Statin therapy in this cohort reflects primary prevention rather than established dyslipidemia or ASCVD. Patients with confirmed dyslipidemia, cardiovascular disease, or other chronic comorbidities were excluded. Occasional antiplatelet use occurred at the discretion of treating physicians and does not indicate chronic comorbidity. Bold values indicate statistically significant differences (p < 0.05)

ACE: Angiotensin-converting enzyme; ARB: Angiotensin receptor blocker; ASCVD: Atherosclerotic cardiovascular disease; BMI: Body mass index; BP: Blood pressure; BSA: Body surface area

^a^Mann–Whitney U test

^b^Student’s t-test

^c^Chi-square test

^d^Fisher’s exact test

### Biochemical and metabolic findings

Biochemical and hematologic variables according to BP control status are summarized in Table 2. Compared with patients with controlled BP, those with uncontrolled BP exhibited impaired renal function (higher serum CRE and lower eGFR), elevated LDH levels, and broader indicators of metabolic stress, including higher uric acid, liver enzyme, and sodium concentrations. These findings suggest a systemic metabolic imbalance associated with suboptimal BP control. The EASIX score was also significantly higher in the uncontrolled BP group, reflecting increased endothelial activation and vascular stress. No significant differences were observed in lipid parameters or inflammatory markers such as CRP and NLR.

**Table 2:** Comparison of biochemical parameters according to BP control status determined by ABPM

Biochemical data	Group		*p*-value
	Controlled BP (n = 76)	Uncontrolled BP (n = 116)	
Routine biochemical parameters			
Fasting glucose (mg/dL), Med (IQR)	89 (15)	90.5 (15)	0.140^c^
Blood urea nitrogen (mg/dL), Med (IQR)	24 (10)	25 (9)	0.324^c^
Creatinine (mg/dL), Med (IQR)	0.69 (0.19)	0.87 (0.21)	** < 0.001**^**c**^
eGFR (mL/min/1.73m^2^), Med (IQR)	100.5 (27)	91 (28)	**0.017**^**c**^
Lactate dehydrogenase (U/L), Med (IQR)	175 (36)	187.5 (59)	**0.012**^**c**^
Sodium (mmol/L), Med (IQR)	139 (3)	140 (3)	**0.002**^**c**^
Potassium (mmol/L), mean ± SD	4.36 ± 0.40	4.33 ± 0.37	0.602^d^
Aspartate transaminase (IU/L), Med (IQR)	17 (7)	19 (9)	**0.011**^**c**^
Alanine transaminase (IU/L), Med (IQR)	17 (11)	21 (14)	**0.013**^**c**^
Albumin (g/dL), Med (IQR)	4.5 (0.5)	4.6 (0.4)	**0.006**^**c**^
Uric acid (mg/dL), Med (IQR)	4.1 (1.4)	5.0 (1.5)	** < 0.001**^**c**^
C-reactive protein (mg/dL), Med (IQR)	1.6 (2.5)	2.1 (3.2)	0.176^**c**^
Triglycerides (mg/dL), Med (IQR)	117 (73)	134.5 (106)	0.061^**c**^
LDL (mg/dL), mean ± SD	116.80 ± 35.25	120.14 ± 34.00	0.513^d^
HDL (mg/dL), Med (IQR)	50 (20)	46 (13)	0.080^**c**^
Total cholesterol (mg/dL), Mean ± SD	194.78 ± 42.21	196.95 ± 42.09	0.727^d^
Complete blood count parameters			
White blood cells (× 10^3^/μL), Med (IQR)	7.24 (2.55)	7.7 (2.41)	0.262^**c**^
Hemoglobin (g/dL), Med (IQR)	13.6 (1.57)	14.7 (2.57)	** < 0.001**^**c**^
Platelet count (× 10⁹/L), Med (IQR)	272 (70)	264.5 (77)	0.066^**c**^
Neutrophil count (× 10^3^/μL), Med (IQR)	4.0 (1.9)	4.43 (1.75)	0.121^**c**^
Lymphocyte count (× 10^3^/μL), Med (IQR)	2.3 (0.9)	2.42 (0.93)	0.648^**c**^
Neutrophil-to-lymphocyte ratio, Med (IQR)	1.7 (1.0)	1.8 (1.0)	0.492^**c**^
EASIX, Med (IQR)	0.44 (0.18)	0.64 (0.31)	** < 0.001**^**c**^
Log_2_ (EASIX), mean ± SD	−1.13 ± 0.44	−0.68 ± 0.51	** < 0.001**^**d**^
SCORE2 risk classification^a^			
Low-risk group [%41 (n = 79)], Med (IQR)	2.1 (1.9)	2.3 (2.0)	0.165^**c**^
Moderate-risk group [%20 (n = 38)], Med (IQR)	4.8 (2.8)	4.6 (4.1)	0.489^**c**^
Patients without calculable risk^b^ [%39 (n = 75)]			

Data are presented as mean ± standard deviation (SD), median (IQR), or number (%), as appropriate. Bold values indicate statistically significant differences (p < 0.05)

ABPM: Ambulatory blood pressure monitoring; BP: Blood pressure; EASIX: Endothelial Activation and Stress Index; eGFR: estimated glomerular filtration rate; SCORE2: Systematic coronary risk evaluation 2

^a^No patients were classified into the high or very high-risk group

^b^SCORE2 risk evaluation does not apply to patients younger than 40 years

^c^Mann–Whitney U test

^d^Student’s t-test

### Ambulatory BP characteristics

Detailed 24-h ABPM parameters are presented in Table 3. Across daytime, nighttime, and 24-h periods, patients with uncontrolled BP consistently demonstrated higher systolic, diastolic, and mean arterial pressures (all p < 0.001). Mean heart rate and pulse pressure were also significantly elevated in the uncontrolled group, further supporting the link between inadequate BP control and increased hemodynamic burden.

**Table 3:** 24-h ABPM parameters by BP control status

24-h ABPM parameters	Group					*p*-value
	Controlled BP (n = 76)			Uncontrolled BP (n = 116)		
Daytime						
Mean systolic BP, mmHg		116.8 ± 8.4	134.0 ± 11.3		** < 0.001**	
Mean diastolic BP, mmHg		72.7 ± 6.5	86.9 ± 9.3		** < 0.001**	
Mean arterial BP, mmHg		93 ± 6.4	108.4 ± 8.9		** < 0.001**	
Mean heart rate, bpm		79.3 ± 10.1	83.2 ± 10.8		**0.011**	
Mean pulse pressure, mmHg		44.2 ± 7.7	47.1 ± 10.5		**0.028**	
Nighttime						
Mean systolic BP, mmHg		106.8 ± 7.9	126.3 ± 12.7		** < 0.001**	
Mean diastolic BP, mmHg		64.0 ± 6.0	79.0 ± 10.4		** < 0.001**	
Mean arterial BP, mmHg		83.6 ± 6.2	100.7 ± 10.4		** < 0.001**	
Mean heart rate, bpm		67.4 ± 8.2	70.8 ± 9.7		**0.011**	
Mean pulse pressure, mmHg		42.8 ± 6.4	47.1 ± 10.0		** < 0.001**	
Overall (24-h)						
Mean systolic BP, mmHg		114.3 ± 7.7	132.1 ± 10.6		** < 0.001**	
Mean diastolic BP, mmHg		70.5 ± 6.0	84.9 ± 9.0		** < 0.001**	
Mean arterial BP, mmHg		90.6 ± 5.8	106.5 ± 8.4		** < 0.001**	
Mean heart rate, bpm		76.3 ± 9.3	80.2 ± 9.9		**0.007**	
Mean pulse pressure, mmHg		43.8 ± 7.2	47.1 ± 9.8		**0.010**	

Data are expressed as mean ± standard deviation (SD). Comparisons were made using the independent t-test. Bold values indicate statistically significant differences (p < 0.05)

ABPM: Ambulatory blood pressure monitoring; BP: Blood pressure

When stratified by nocturnal dipping status, non-dippers (n = 121) showed slightly higher EASIX and log₂ (EASIX) values compared with dippers (n = 71); however, these differences were not statistically significant (EASIX: 0.54 [IQR 0.32] vs. 0.51 [IQR 0.31], p = 0.143; log_2_ [EASIX]: −0.81 ± 0.51 vs. −0.93 ± 0.55, p = 0.158; Supplementary Table S1).

### Regression analysis

In the univariate analyses (Table 4), several clinical, echocardiographic, and laboratory variables were associated with uncontrolled BP. All variables meeting the p < 0.10 threshold were considered eligible for inclusion in the multivariable logistic regression model. Before constructing the final model, these candidate predictors were identified using appropriate univariate comparisons based on 24-h ABPM–defined BP control status.

**Table 4:** Univariate analysis of clinical, echocardiographic, and laboratory variables by BP control status

Variable	p-value
Age	0.816^a^
Sex	** < 0.001**^**c**^
Height	** < 0.001**^**a**^
Weight	** < 0.001**^**b**^
Body surface area	** < 0.001**^**b**^
Interventricular septal thickness	** < 0.001**^**a**^
Left ventricular posterior wall thickness	** < 0.001**^**a**^
Left ventricular end-diastolic diameter	** < 0.001**^**a**^
Ascending aortic diameter	** < 0.001**^**a**^
Left atrial diameter	** < 0.001**^**b**^
Left ventricular mass index	** < 0.001**^**b**^
Relative wall thickness	**0.037**^**b**^
Serum creatinine	** < 0.001**^**a**^
Estimated glomerular filtration rate	**0.017**^**a**^
Lactate dehydrogenase	**0.012**^**a**^
Serum sodium	**0.002**^**a**^
Aspartate aminotransferase	**0.011**^**a**^
Alanine aminotransferase	**0.013**^**a**^
Serum albumin	**0.006**^**a**^
Uric acid	** < 0.001**^**a**^
Hemoglobin	** < 0.001**^**a**^
EASIX	** < 0.001**^**a**^
Log_2_ (EASIX)	** < 0.001**^**b**^

BP: Blood pressure; EASIX: Endothelial Activation and Stress Index

p-values were calculated using appropriate statistical tests. Bold values indicate statistically significant differences (p < 0.05)

^a^Mann–Whitney U test

^b^Student’s t-test

^c^Chi-square test

These variables were then evaluated for linear relationships with log_2_ (EASIX) using Pearson correlation analysis. None demonstrated a very strong correlation (|r|≥ 0.7). Subsequently, linear regression diagnostics were performed to assess multicollinearity among the candidate predictors using VIFs. Variables with VIF values > 10 were considered collinear and were excluded from further modeling to maintain statistical validity.

Based on the multicollinearity assessment, height, weight, body surface area, interventricular septal thickness, left ventricular posterior wall thickness, left ventricular end-diastolic diameter, left ventricular mass index, mean arterial pressure, and serum CRE were removed from further analysis. Additionally, biochemical components that directly contribute to the EASIX formula (LDH, CRE, and derived eGFR) were excluded to avoid redundancy and circular associations within the regression model.

As shown in Table 5, log_2_ (EASIX), ascending aortic diameter, serum sodium, and serum albumin remained independent predictors of uncontrolled BP after adjustment for age and sex. The strongest association was observed for log_2_ (EASIX) (OR 6.31; 95% CI 2.56–15.52; p < 0.001), followed by ascending aortic diameter (OR 1.24; 95% CI 1.09–1.41; p = 0.001), serum albumin (OR 5.77; 95% CI 1.58–21.14; p = 0.008), and serum sodium (OR 1.19; 95% CI 1.02–1.38; p = 0.029).

**Table 5:** Logistic regression analysis of factors associated with BP control status

Variable	B	SE	Wald	p-value	OR	95% CI for OR (Lower – Upper)
Age	−0.006	0.017	0.124	0.725	0.994	0.962–1.027
Sex (male)	0.270	0.577	0.220	0.639	1.311	0.423–4.060
Ascending aorta	**0.210**	**0.072**	**8.457**	**0.004**	**1.234**	**1.071–1.422**
Left atrium	0.017	0.060	0.082	0.775	1.017	0.904–1.145
Serum sodium	**0.161**	**0.079**	**4.130**	**0.042**	**1.175**	**1.006–1.372**
AST	0.012	0.036	0.116	0.733	1.012	0.943–1.086
ALT	0.022	0.021	1.114	0.291	1.022	0.981–1.065
Serum albumin	**2.022**	**0.697**	**8.421**	**0.004**	**7.555**	**1.928–29.611**
Uric acid	0.114	0.172	0.442	0.506	1.121	0.801–1.570
Hemoglobin	0.149	0.128	1.361	0.243	1.161	0.903–1.492
Log_2_ (EASIX)	**1.634**	**0.494**	**10.942**	** < 0.001**	**5.126**	**1.946–13.500**
Constant	**−40.073**	**12.081**	**11.003**	** < 0.001**	**0.000**	**—**

Data are presented as β coefficients (standard error [SE]), odds ratios (OR) with 95% confidence intervals (CI), and p-values from multivariable logistic regression analysis. Rows shown in bold indicate variables that remained statistically significant in the multivariable regression model (p < 0.05)

ALT: Alanine aminotransferase; AST: Aspartate aminotransferase; EASIX: Endothelial Activation and Stress Index; BP: Blood pressure

To construct a parsimonious model, the multivariable logistic regression was ultimately fitted using only the variables that remained significant after adjustment—ascending aortic diameter, serum sodium, serum albumin, and log_2_ (EASIX). A simplified presentation of this final model is provided in Supplementary Table S2. Model diagnostics demonstrated good calibration (Hosmer–Lemeshow χ^2^ = 6.77, p = 0.562) and acceptable explanatory power (Nagelkerke R^2^ = 0.40). The model correctly classified 77.6% of patients overall (74.1% with controlled BP and 80.2% with uncontrolled BP).

### ROC curve analysis

**Fig. 2:**
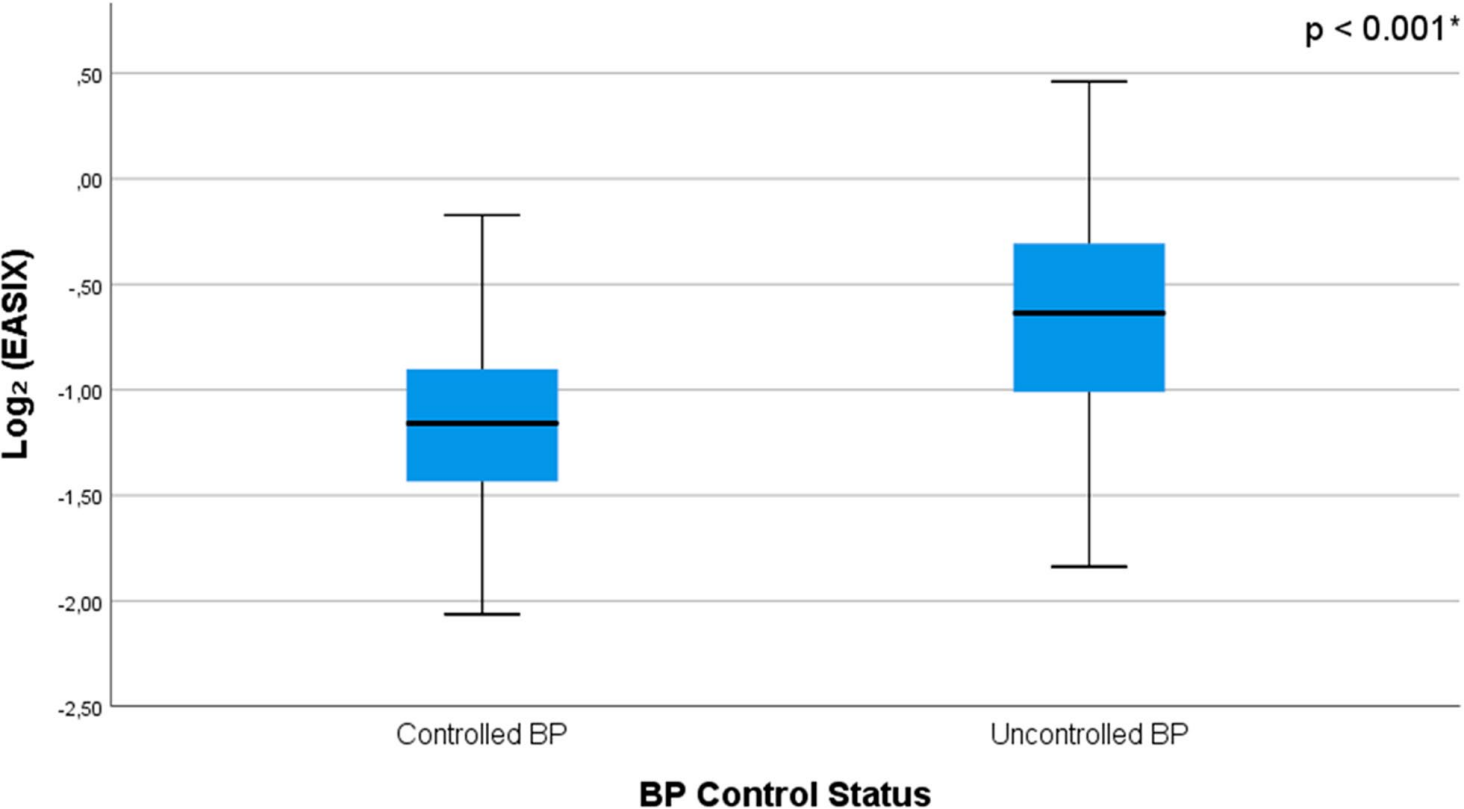
ROC curves comparing the discriminative ability of independent predictors identified in the logistic regression model

To assess the discriminative performance of EASIX and the other independent variables, ROC analyses were conducted for each parameter (Fig. 2, Table 6). Log_2_ (EASIX) demonstrated the highest AUC (0.755, 95% CI 0.685–0.825), followed by ascending aortic diameter (AUC = 0.684, 95% CI 0.606–0.762), serum sodium (AUC = 0.633, 95% CI 0.553–0.714), and serum albumin (AUC = 0.617, 95% CI 0.534–0.700).

**Table 6:** ROC analysis of independent predictors of BP control status

Variable	Area (AUC)	Std Error^a^	Asymptotic Sig.^b^	95% CI	
				Lower Bound	Upper Bound
Serum sodium	0.633	0.041	0.002	0.553	0.714
Serum albumin	0.617	0.042	0.006	0.534	0.700
Log_2_ (EASIX)	**0.755**	**0.036**	** < 0.001**	**0.685**	**0.825**
Ascending aorta	0.684	0.040	< 0.001	0.606	0.762

Data are presented as area under the curve (AUC) with corresponding standard error (SE) and 95% confidence interval (CI). Statistical significance was assessed using the nonparametric assumption

ROC: Receiver operating characteristic

^a^Under the nonparametric assumption

^b^Null hypothesis: true area = 0.5

Optimal discriminative thresholds were identified using the Youden index, yielding a cut-off of 0.48 for EASIX and –1.05 for log_2_ (EASIX), corresponding to 80% sensitivity and 68% specificity.

**Fig. 3:**
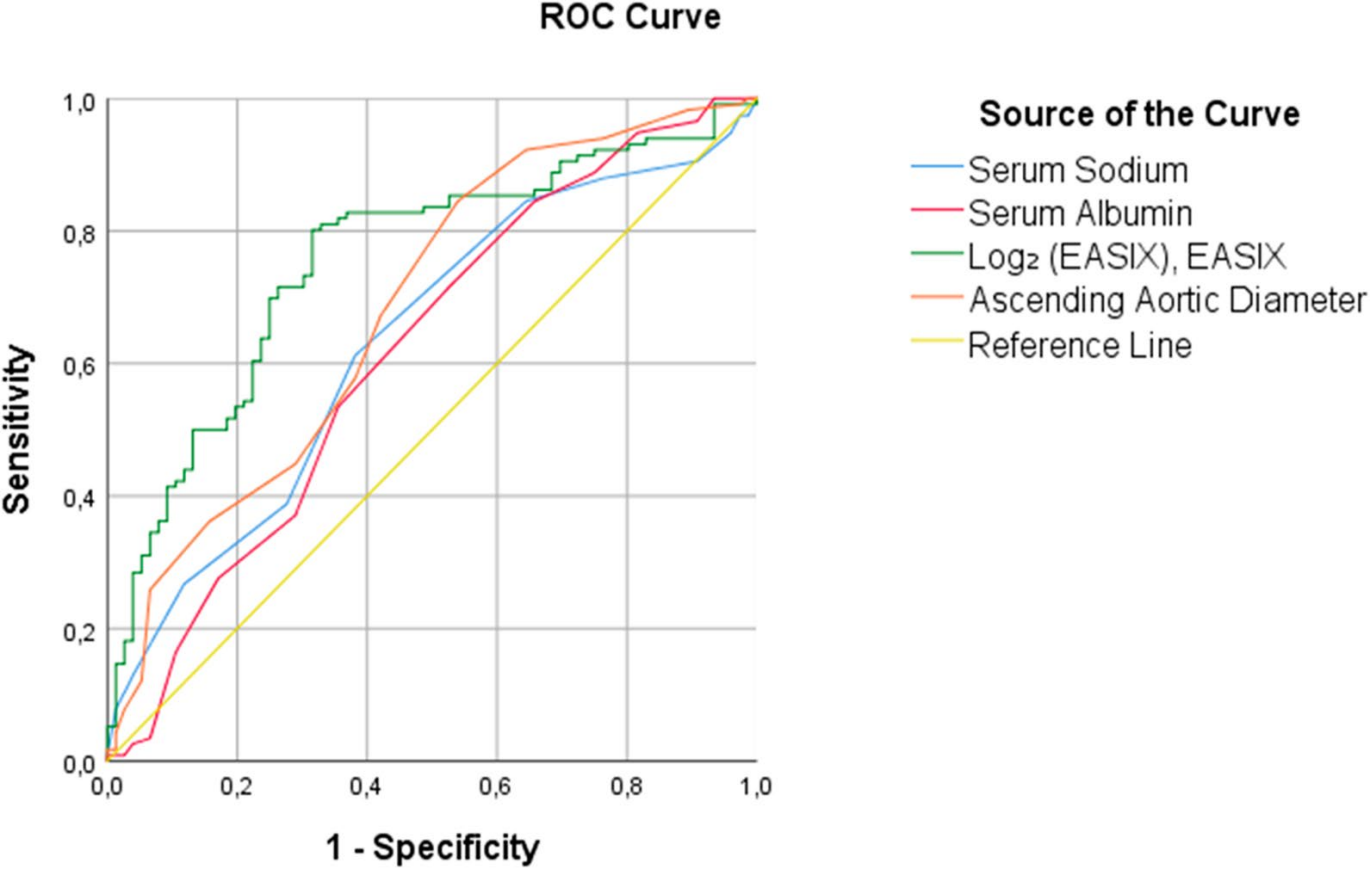
Comparison of EASIX scores by BP control status as determined by ABPM

As shown in Fig. 3, patients with uncontrolled BP exhibited significantly higher EASIX scores (p < 0.001), reinforcing the association between elevated EASIX values and inadequate BP control.

## Discussion

This study demonstrated that patients with uncontrolled BP, as determined by 24-h ABPM, had significantly higher EASIX values compared with those with controlled BP. Among the evaluated parameters, log₂ (EASIX), ascending aortic diameter, serum sodium, and serum albumin emerged as independent factors associated with BP control status. These findings suggest that EASIX captures vascular and endothelial stress that parallels inadequate BP regulation.

Endothelial dysfunction is a well-established contributor to HTN pathophysiology [18–20]. The present findings are consistent with these mechanisms, suggesting that higher EASIX values may reflect ongoing subclinical endothelial stress in patients with inadequate BP control. Inflammatory and metabolic markers—including CRP, uric acid, and the NLR—were also evaluated but showed no significant association with BP control status. In contrast, EASIX, derived from routine laboratory parameters (LDH, CRE, and PLT count), demonstrated a stronger association, underscoring its potential clinical relevance. Other endothelial biomarkers such as asymmetric dimethylarginine (ADMA), vascular cell adhesion molecule-1 (VCAM-1), and flow-mediated dilation (FMD) could not be assessed due to the retrospective design and their absence in routine hypertensive evaluations [21]. From a cost-effectiveness standpoint, EASIX offers a practical advantage because it relies solely on laboratory tests that are routinely obtained in everyday clinical practice.

Previous studies have expanded the application of EASIX beyond hematologic disorders into various cardiovascular settings. In a nationwide hypertensive cohort, Dong et al. (2025) reported a significant association between EASIX and both cardiovascular and all-cause mortality [22]. However, their analysis relied on office-based BP measurements and included patients with multiple comorbidities. To our knowledge, no prior study has examined the relationship between EASIX and ambulatory BP control in a strictly defined cohort with isolated primary HTN. Accordingly, our findings extend the current evidence by addressing this clinically relevant and methodologically homogeneous population.

Additional research supports the prognostic role of EASIX across cardiovascular disease states. Yin and Wang (2025) linked elevated log_2_ (EASIX) values with one-year mortality in heart failure patients [23], while Krombholz-Reindl et al. (2025) demonstrated its predictive value for long-term outcomes following coronary artery bypass grafting [24]. Likewise, Xia et al. (2025) observed higher mortality in atrial fibrillation patients with elevated EASIX quartiles [25]. Taken together, these findings support the role of EASIX as a practical indicator of systemic and endothelial stress.

In the present analysis, ascending aortic diameter, serum sodium, serum albumin, and log_2_ (EASIX) emerged as independent predictors of BP control. The relationship between aortic dilation and HTN is well-documented, likely involving both hemodynamic and structural determinants [26–28]. Elevated serum sodium, though a weak predictor, aligns with prior evidence linking sodium balance to endothelial dysfunction [29–32]. Similarly, Xu et al. (2023) reported differences in sodium levels across EASIX strata [33].

The role of serum albumin in HTN remains debated: while some studies report a positive correlation [34–36], others suggest an inverse relationship [37–39]. In our cohort, albumin was a significant but complex determinant, potentially reflecting interactions between vascular permeability, plasma oncotic pressure, and endothelial integrity.

### Study limitations

This study has several limitations. Its retrospective, single-center design introduces the possibility of selection bias and residual confounding. Although patients with major comorbidities were excluded, mild hepatic or renal variations—known to influence LDH, CRE, and PLT levels—may still have affected EASIX values. Medication adherence, duration of HTN, and treatment intensity were not fully captured and may also impact BP control. Because both BP control status and EASIX were assessed at a single time point, it is not possible to determine whether elevated EASIX values contribute to uncontrolled BP or predominantly reflect the downstream consequences of prolonged hypertension and related metabolic stress. The logistic regression model, while adjusted for key variables, was not externally validated, and the ROC-derived thresholds should not be interpreted as clinically definitive. Finally, the findings apply only to patients without comorbidities and may not extend to broader hypertensive populations.

## Conclusions

In this cohort of patients with isolated primary HTN, higher EASIX values were associated with uncontrolled BP as determined by 24-h ABPM. While EASIX demonstrated moderate discriminative ability, it should currently be regarded as an exploratory biomarker reflecting endothelial and metabolic stress rather than a predictive tool. Prospective, multicenter studies with longitudinal follow-up are needed to determine whether EASIX provides incremental clinical value in HTN management.

## Supplementary Information

Supplementary Material 1. Supplementary Figure S1. Distribution of EASIX values before and after log_2_ transformation. Histograms illustrate normalization of the right-skewed raw EASIX distribution following log_2_ transformation, supporting the use of log-transformed values in regression and ROC analyses. EASIX : Endothelial activation and stress index; ROC : Receiver operating characteristic.

Supplementary Material 2. Supplementary Table S1. Comparison of EASIX and log_2_values between dipper and non-dipper patients. Data are presented as medianor mean ± standard deviationas appropriate. Mann–Whitney U test was used for non-normally distributed variablesand independent samples t-test for normally distributed variables. EASIX : Endothelial activation and stress index.

Supplementary Material 3. Supplementary Table S2. Simplified Logistic Regression Analysis of Factors Associated with Blood Pressure Control Status. Data are presented as β coefficients, odds ratioswith 95% confidence intervals, and p-values from multivariable logistic regression analysis. EASIX : Endothelial activation and stress index

## Data Availability

The datasets used and/or analysed during the current study are available from the corresponding author on reasonable request.

## Abbreviations

ABPM: Ambulatory blood pressure monitoring
ADMA: Asymmetric dimethylarginine
ASE: American society of echocardiography
AUC: Area under the curve
aGVHD: Acute graft-versus-host disease
BMI: Body mass index
BP: Blood pressure
CRE: Creatinine
CRP: C-reactive protein
EASIX: Endothelial activation and stress index
ESC: European society of cardiology
FMD: Flow-mediated dilation
HTN: Hypertension
LDH: Lactate dehydrogenase
NLR: Neutrophil-to-lymphocyte ratio
PLT: Platelet
ROC: Receiver operating characteristic
VCAM-1: Vascular cell adhesion molecule-1
VIF: Variance inflation factor
