# Supplementary figures and images for: The role of the EASIX score in patients with hypertension: a cross-sectional study
Source: Egypt Heart J. 2025 Dec 24;77:112. doi: 10.1186/s43044-025-00710-7 (PMC12738477; doi:10.1186/s43044-025-00710-7)

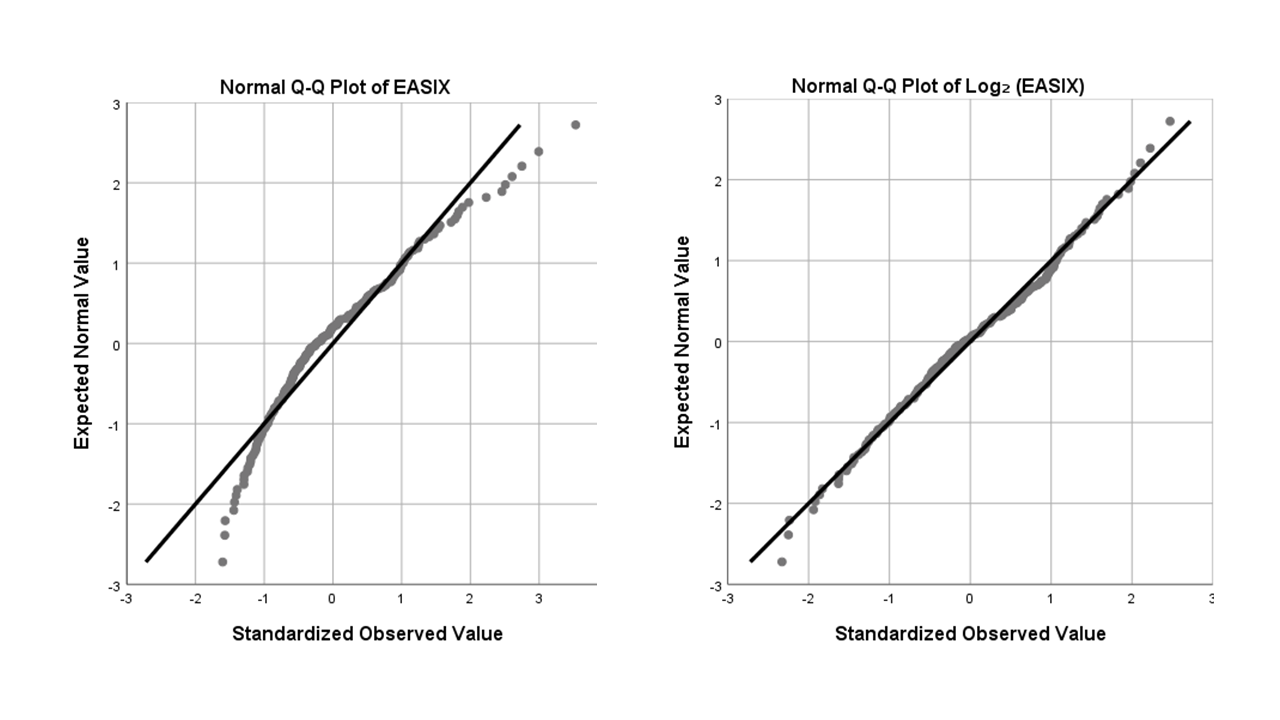

Supplement: Supplementary file 1 — Supplementary Material 1. Supplementary Figure S1. Distribution of EASIX values before and after log2 transformation. Histograms illustrate normalization of the right-skewed raw EASIX distribution following log2 transformation, supporting the use of log-transformed values in regression and ROC analyses. EASIX : Endothelial activation and stress index; ROC : Receiver operating characteristic. [file 43044_2025_710_MOESM1_ESM.png]
