# Supplementary material for: The role of the EASIX score in patients with hypertension: a cross-sectional study
Source: Egypt Heart J. 2025 Dec 24;77:112. doi: 10.1186/s43044-025-00710-7 (PMC12738477; doi:10.1186/s43044-025-00710-7)
Supplement: Supplementary file 2 — Supplementary Material 2. Supplementary Table S1. Comparison of EASIX and log2values between dipper and non-dipper patients. Data are presented as medianor mean ± standard deviationas appropriate. Mann–Whitney U test was used for non-normally distributed variablesand independent samples t-test for normally distributed variables. EASIX : Endothelial activation and stress index. [file 43044_2025_710_MOESM2_ESM.docx]

# Supplementary Table S1. Comparison of EASIX and log₂ (EASIX) values between dipper and non-dipper subgroups

| **Parameter** | **Dipper**  **(n = 71)** | **Non-Dipper**  **(n = 121)** | **p-value** |
| --- | --- | --- | --- |
| EASIX, Median (IQR) | 0.51 (0.31) | 0.54 (0.32) | 0.143* |
| log₂ (EASIX), Mean ± SD | –0.93 ± 0.55 | –0.81 ± 0.51 | 0.158† |

Data are presented as median (IQR) or mean ± standard deviation (SD) as appropriate. Mann–Whitney U test was used for non-normally distributed variables (*) and independent samples t-test for normally distributed variables (†). Abbreviations: EASIX = endothelial activation and stress index.
