# Supplementary material for: The role of the EASIX score in patients with hypertension: a cross-sectional study
Source: Egypt Heart J. 2025 Dec 24;77:112. doi: 10.1186/s43044-025-00710-7 (PMC12738477; doi:10.1186/s43044-025-00710-7)
Supplement: Supplementary file 3 — Supplementary Material 3. Supplementary Table S2. Simplified Logistic Regression Analysis of Factors Associated with Blood Pressure Control Status. Data are presented as β coefficients, odds ratioswith 95% confidence intervals, and p-values from multivariable logistic regression analysis. EASIX : Endothelial activation and stress index [file 43044_2025_710_MOESM3_ESM.docx]

| **Variable** | **B** | **S.E.** | **Wald** | **p-value** | **OR** | **95% CI for OR (Lower – Upper)** |
| --- | --- | --- | --- | --- | --- | --- |
| Age | -0.006 | 0.016 | 0.153 | 0.696 | 0.994 | 0.962 – 1.026 |
| Sex (male) | -0.212 | 0.500 | 0.180 | 0.671 | 0.809 | 0.304 – 2.155 |
| **Ascending aorta** | **0.214** | **0.066** | **10.597** | **<0.001** | **1.238** | **1.089 – 1.408** |
| **Serum sodium** | **0.170** | **0.078** | **4.755** | **0.029** | **1.186** | **1.017 – 1.382** |
| **Serum albumin** | **1.753** | **0.663** | **6.997** | **0.008** | **5.769** | **1.575 – 21.141** |
| **Log₂ (EASIX)** | **1.841** | **0.460** | **16.046** | **<0.001** | **6.305** | **2.561 – 15.521** |
| **Constant** | **-35.909** | **11.651** | **9.499** | **0.002** | **0.000** | **—** |

**Supplementary Table S2.** Simplified Logistic Regression Analysis of Factors Associated with Blood Pressure Control Status

*Data are presented as β coefficients (standard error [SE]), odds ratios (OR) with 95% confidence intervals (CI), and p-values from multivariable logistic regression analysis. Abbreviations: EASIX = endothelial activation and stress index.*
